# Bone Metabolism and Vitamin D Implication in Gastroenteropancreatic Neuroendocrine Tumors

**DOI:** 10.3390/nu12041021

**Published:** 2020-04-08

**Authors:** Barbara Altieri, Carla Di Dato, Roberta Modica, Filomena Bottiglieri, Antonella Di Sarno, James F.H. Pittaway, Chiara Martini, Antongiulio Faggiano, Annamaria Colao

**Affiliations:** 1 Division of Endocrinology and Diabetes, Department of Internal Medicine I, University Hospital, University of Wuerzburg, 97080 Wuerzburg, Germany; 2 Department of Clinical Medicine, Bufalini Hospital, 47521 Cesena, Italy; didatocarla@gmail.com; 3 Department of Clinical Medicine and Surgery, Federico II University, 80131 Naples, Italy; robertamodica@libero.it (R.M.); lenabottiglieri@gmail.com (F.B.); colao@unina.it (A.C.); 4 UOC of Internal Medicine, AO dei Colli, Monaldi Unit, 80131 Naples, Italy; antonella.disarno@gmail.com; 5 Centre for Endocrinology, William Harvey Research Institute, Barts and the London School of Medicine and Dentistry, Queen Mary University of London, EC1M 6BQ London, UK; jfhpittaway@doctors.org.uk; 6 Clinica Medica 3, Department of Medicine, DIMED, University of Padova, 35128 Padova, Italy; chiaramartini56@gmail.com; 7 Department of Experimental Medicine, Sapienza University of Rome, 00161 Rome, Italy; antongiulio.faggiano@uniroma1.it

**Keywords:** bone, vitamin D, neuroendocrine tumor, osteoporosis, mineral bone density, cortisol, serotonin, miRNA, MEN1, therapy

## Abstract

Patients affected by gastroenteropancreatic–neuroendocrine tumors (GEP–NETs) have an increased risk of developing osteopenia and osteoporosis, as several factors impact on bone metabolism in these patients. In fact, besides the direct effect of bone metastasis, bone health can be affected by hormone hypersecretion (including serotonin, cortisol, and parathyroid hormone-related protein), specific microRNAs, nutritional status (which in turn could be affected by medical and surgical treatments), and vitamin D deficiency. In patients with multiple endocrine neoplasia type 1 (MEN1), a hereditary syndrome associated with NET occurrence, bone damage may carry other consequences. Osteoporosis may negatively impact on the quality of life of these patients and can increment the cost of medical care since these patients usually live with their disease for a long time. However, recommendations suggesting screening to assess bone health in GEP–NET patients are missing. The aim of this review is to critically analyze evidence on the mechanisms that could have a potential impact on bone health in patients affected by GEP–NET, focusing on vitamin D and its role in GEP–NET, as well as on factors associated with MEN1 that could have an impact on bone homeostasis.

## 1. Introduction

Bone is a highly dynamic organ that undergoes significant constant remodeling. This allows self-repair, for example, after a fracture, and to adapt to forces placed on it [1]. In physiological conditions, bone health derives from the balance between osteoblastic bone formation and osteoclastic bone resorption. These processes determine structural integrity and govern the risk of fractures [2]. Disturbance of the balance between bone formation and resorption causes different skeletal disorders, including osteoporosis. Osteoporosis constitutes a major public health problem and is characterized by low bone mass and microarchitectural deterioration of bone tissue, leading to bone fragility and increased fracture risk [3,4]. Osteoporotic vertebral and hip fractures are associated with severe and chronic pain, decreased mobility, activity restriction and reduced ability to carry out daily activities, with a significant impact on the individual’s health-related quality of life (HRQoL) that accumulates over time [5,6]. Furthermore, back pain is experienced by osteoporotic patients also in absence of vertebral fracture [7], and could negatively impact HRQoL, walking speed, balance and leg strength in women with and without vertebral fractures [8].

Malignant neoplasms are associated with a deterioration of bone health, resulting in a major risk of skeletal complications, bone pain, and worsened quality of life. This condition can be connected to the development of bone metastasis, pharmacological treatment, and nutritional status [9]. Neuroendocrine tumors (NET) are a heterogeneous group of neoplasms that can arise in any tissue and organ, although the majority of these tumors affect the gastroenteropancreatic (GEP) tract [10]. The frequency of NET is reported to be increasing in a number of countries over the last few decades [11,12]. Bone metastasis are reported in 4–12% of NET patients, but the real incidence is probably higher [13,14,15,16]. However, except for those patients who have a metastatic disease associated with shorter survival [13], patients with NET usually have good long-term survival [10,17]. It has been demonstrated that NET patients have a greater clinical burden of disease than matched control subjects, with increased risk of cardiovascular, hepatic, and gastrointestinal diseases, as well as osteopenia and osteoporosis [18]. Moreover, the tumor itself and the systemic therapy may have an impact on patients’ nutritional status [19,20], leading to a deficiency of different vitamins, including vitamin D [21,22]. It is well demonstrated that vitamin D plays a key role in the process of bone formation [23]. Thus, vitamin D deficiency observed in NET patients could also be implicated in the deterioration of bone, promoting osteopenia and osteoporosis. Thus, bone health may be altered in NET, independently from the development of metastasis. However, data from literature are very scant and it is difficult to understand mechanisms affecting bone metabolism in NET. 

The aim of this review is to critically analyze evidence on mechanisms that could potentially damage bone health in patients affected by GEP–NET and to discuss the clinical implications for the management of these patients. We also extensively evaluated the role of vitamin D in GEP–NET patients and factors associated with multiple endocrine neoplasia type 1 (MEN1) syndrome that could have an impact on bone metabolism. We decided to focus on GEP–NET because, in our opinion, the clinical characteristics and treatments available make these kinds of neoplasms more susceptible to developing disturbances of bone metabolism.

## 2. Mechanisms of Bone Damage in Functional and Non-Functional GEP–NET

**Figure 1:**
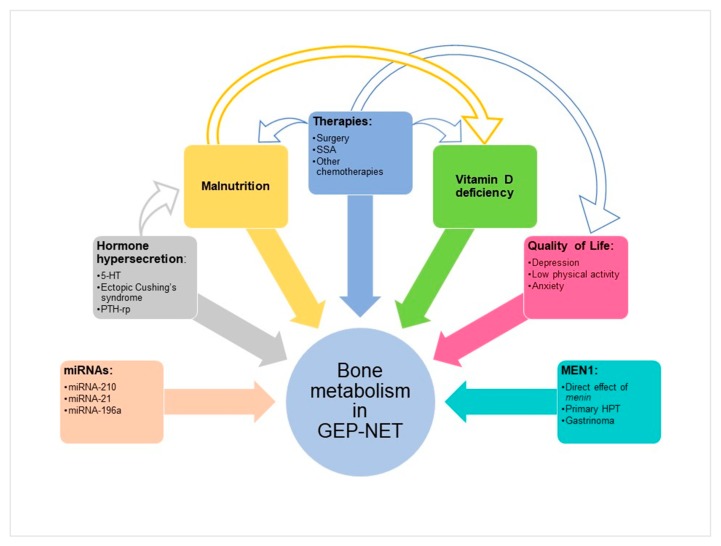
Factors that have an impact on bone metabolism in gastroenteropancreatic–neuroendocrine tumors (GEP–NETs) patients in addition to bone metastases. The arrows indicate the interaction among the different factors.

A higher prevalence of osteoporosis and osteopenia has been reported in NET patients, and for those younger than 50 presents with an increased risk of developing osteopenia or osteoporosis compared to the matched control group (odds ratio (OR) = 3.24, 95% CI: 1.36–7.73, *p* = 0.008) [18]. The exact mechanism of this increased risk is not entirely known. Besides bone metastasis, different factors could have an impact on bone health in GEP–NET patients, including hormone hypersecretion, specific micro-RNAs (miRNAs), nutritional status, vitamin D deficiency, quality of life, and aspects correlated to MEN1 (Figure 1). 

Principal clinical studies investigating bone metabolism and vitamin D status in GEP–NET patients that will be discussed in detail in this review are summarized in Table 1. 

### 2.1. Hormone Hypersecretion

**Figure 2:**
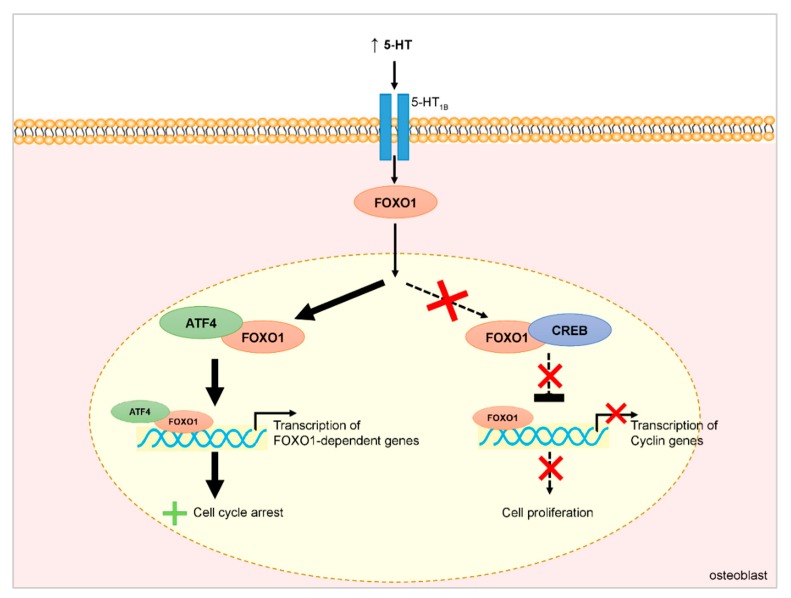
Effect of serotonin (5-HT) on bone metabolism under pathological conditions, such as carcinoid syndrome: the association between forkhead box protein O1 (FOXO1) and cAMP-responsive element–binding protein 1 (CREB) is disrupted, whereas the formation of activating transcription factor 4 (ATF4)-FOXO1 heterodimers is favored. Subsequently, the transcription mediated by FOXO1 is not inhibited, leading to the expression of FOXO1-dependent genes that mediate cell cycle arrest.

GEP–NETs can be associated with functional syndromes. The most frequent functional syndrome associated with NET is carcinoid syndrome (CS). Over 40 humoral substances have been identified as being potentially involved in the pathogenesis of carcinoid syndrome, but the main mediator is serotonin (5-HT) [35]. 5-HT is also one of the key players in bone tissue dynamics [36]. It has been demonstrated that osteoblasts, osteoclasts, and osteocytes express tryptophan hydroxylase 1 (TPH1), which synthetizes 5-HT, 5-HT transporters (SERT), and 5-HT receptors [37,38]. Depending on the site of production, 5-HT can exert contrasting effects on bone density. When produced in the brain, 5-HT exerts a positive effect on bone mass by decreasing the inhibition of sympathetic nerves on osteoblasts and consequently enhancing bone formation. On the contrary, 5-HT secreted by the neuroendocrine cells of the gastrointestinal tract inhibits osteoblast proliferation by its binding to 5-HT_1B_ receptors expressed by pre-osteoblasts [39,40]. The role played by gut-derived 5-HT in the regulation of bone metabolism has been further defined through a link between a member of the low-density lipoprotein receptor (Lrp) family, Lrp5, and the level of TPH1 in the neuroendocrine cells of the gastrointestinal tract [41]. It has been demonstrated that Lrp5 knockout mice had increased TPH1 expression on neuroendocrine cells of the gastrointestinal tract, increased levels of circulating 5-HT, and decreased bone density [41]. At the molecular level, the action of 5-HT on osteoblasts is mediated by the transcription factor forkhead box protein O1 (FOXO1). Under physiological conditions, FOXO1 interacts with both cAMP-responsive element–binding protein 1 (CREB) and activating transcription factor 4 (ATF4) in the nucleus of osteoblasts (Figure 2) [42]. The interaction with ATF4 promotes the transcriptional activity of FOXO1 and the expression of FOXO1-dependent genes, which mediate cell cycle arrest, whereas the interaction with CREB suppresses FOXO1 transcription and stimulates the expression of cyclin genes, which mediate cell proliferation. The balance of the opposing actions of these two transcription factors maintains normal osteoblast proliferation. In pathological conditions of high 5-HT levels, as observed in carcinoid syndrome, the association between FOXO1 and CREB is disrupted, favoring the formation of ATF4-FOXO1 heterodimers that resulted in an upregulation of the ATF4-mediated responses (Figure 2). These events subsequently lead to suppression of cyclin genes and cell cycle arrest [42].

The effect of 5-HT as a possible mediator in bone damage was also confirmed by several preclinical and clinical studies showing that selective serotonin reuptake inhibitors are associated with bone loss and an increase in fracture risk [41,43]. However, clinical studies in NET patients did not confirm the alteration of bone metabolism in the condition of high levels of 5-HT. Two different studies demonstrated that bone mineral density (BMD), as well as markers of bone formation and markers of bone resorption, were not different between patients with high and low urinary 5-hydroxy-indoleacetic acid (5-HIAA) levels, the 5-HT metabolite (Table 1) [24,25]. Another study comparing 26 midgut NET with carcinoid syndrome and matched healthy controls showed that high circulating 5-HT levels in carcinoid syndrome were not associated with lower BMD, poorer bone structure, or lower levels of bone formation markers (Table 1) [26].

Besides carcinoid syndrome, functional pancreatic NET (pNET) have to be mentioned. The two most common functional pNET are gastrinoma and insulinoma, followed by VIPoma, glucagonoma, GRFoma, ACTHoma (leading to ectopic Cushing’s syndrome, ECS), tumors causing carcinoid syndrome or hypercalcemia (PTHrPomas), and somatostatinoma. In addition to these, there are also very rare functional pNET secreting renin, luteinizing hormone (LH), erythropoietin, glucagon-like peptide-1(GLP-1), insulin-like growth factor-2 (IGF-II), and cholecystokinin (CCKoma). Non-functional p-NET are not associated with specific symptoms but frequently secrete pancreatic polypeptide, chromogranin A, neuron-specific enolase, human chorionic gonadotrophin subunits, calcitonin, neurotensin, or other peptides [12,44]. To our knowledge, there are no specific studies about functional and non-functional pNETs and bone health. Nevertheless, ECS caused by ACTH secretion and primary hyperparathyroidism (HPT) is already recognized as a cause of secondary osteoporosis [45,46]. Gastrinoma will be discussed later within MEN1. 

**Figure 3:**
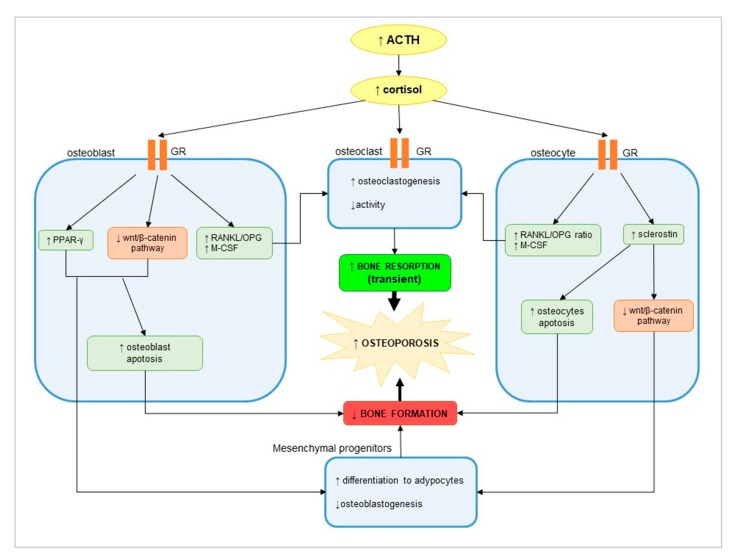
Direct effect of cortisol excess due to ectopic ACTH secretion on bone health: osteoblasts, osteocytes, and osteoclasts express glucocorticoid receptors (GRs), which mediate the cortisol action. Cortisol excess induces the secretion of peroxisome proliferator-activated receptor (PPAR)-γ and inhibits the wnt pathway, inducing osteoblast apoptosis and differentiation of mesenchymal progenitors into adipocytes. On the other side, Sclerostin induces osteocyte apoptosis and inhibits the wnt pathway. These mechanisms inhibit bone formation. The increase of the receptor activator for NF-κB ligand (RANKL)/osteoprotegerin (OPG) ratio, together with the increased macrophage colony-stimulating factor (M-CSF), stimulates osteoclastogenesis and bone resorption. Decrease of bone formation and increase of bone resorption leads to osteopenia/osteoporosis.

ECS represents only 10% of all causes of Cushing’s, and among these pNET, represents only a small percentage of cases. However, ECS is the most frequent endocrine paraneoplastic syndrome reported in NET [47]. It has well demonstrated that cortisol excess has a negative impact on bone health [48,49,50]. Osteoporosis induced by glucocorticoid excess is due mainly to a direct effect on osteoblasts, osteocytes, and osteoclasts, which express glucocorticoid receptors (GR; Figure 3) [49]. In osteoblasts, cortisol excess induces a reduction of bone formation mediated by an upregulation of peroxisome proliferator-activated receptor (PPAR)-γ and an inhibition of the wnt pathway, which promotes, in turn, osteoblast apoptosis and differentiation of mesenchymal progenitors into adipocytes. These processes result in a low number of osteoblasts and decreasing bone formation [51]. Sclerostin secreted by osteocytes induces osteocyte apoptosis and inhibits the wnt pathway. Moreover, the increase of the receptor activator for NF-κB ligand (RANKL)/osteoprotegerin (OPG) ratio, together with the increased macrophage colony-stimulating factor (M-CSF), stimulates osteoclastogenesis and bone resorption (Figure 3) [49]. 

In addition to this direct effect on bone, glucocorticoid excess may also have a negative impact on vitamin D levels [52]. Different case reports describe the occurrence of osteoporosis in patients with GEP–NET and ECS (Table 1) [27,28]. Interestingly, a 34-year-old female patient with a history of multiple spontaneous rip fractures and osteoporosis, who had been diagnosed with NET of the appendix associated with ECS, reported an increase in BMD during the three years of follow-up after tumor resection [28]. This study represents a potential reversibility of steroid-induced osteoporosis and, when possible, complete resection of the primary tumors should be considered in the management of such patients.

Similarly, hypersecretion of parathyroid hormone (PTH)-related protein determines, with different mechanisms, an imbalance between bone resorption (normal or increased, especially in the early phase) and bone formation (impaired, particularly in the chronic phase), with a consequent increased risk of fractures [53].

### 2.2. MicroRNAs

MiRNAs are small noncoding single-stranded RNAs of 21–23 nucleotides long that regulate the expression of 1%–4% of human genes at a posttranscriptional level. Therefore, miRNAs are involved in many physiological and pathological conditions, including GEP–NET progression and metastatic spread [13,54]. 

Several miRNAs are involved in the regulation of bone metabolism and homeostasis. However, in pathological conditions, impaired miRNA signaling could contribute to the onset of skeletal disorders, including osteoporosis [55]. Among miRNAs that regulate osteoblast differentiation [56,57], miRNA-210, miRNA-21, and mi-RNA 196a may play key roles in the regulation of bone metabolism in patients affected by GEP–NET (Figure 1). It has been demonstrated that miRNA-210 enhances the differentiation of bone marrow mesenchymal stem cells into osteoblasts in addition to inducing the expression of vascular endothelial growth factors [58]. Moreover, miRNA-210 is upregulated in GEP–NET and is associated with metastatic disease, but not with tumor grade (G) [59]. Also, miRNA-21, which is overexpressed in pNET patients with high Ki67 proliferation index (>2) and presence of liver metastasis [60], plays a crucial role in osteoblast differentiation, enhancing the expression of matrix metallopeptidases and promoting epithelial to mesenchymal transition [61]. Finally, miRNA-196a regulates the osteogenic differentiation and proliferation of human adipose tissue-derived mesenchymal stem cells [62]. It has been shown that high expression of miRNA-196a is significantly associated with pNET having aggressive biological behavior, characterized by advanced stage, lymph node metastases, higher mitotic count and higher Ki67 index (≥3%, corresponding to G2–G3 tumor), and worse clinical outcomes [63]. 

However, the potential role of these or other miRNAs in the maintenance of bone metabolism in patients with GEP–NET has not been directly investigated. Therefore, specific studies are needed to better elucidate this topic.

### 2.3. Nutritional Status

Bone health is strictly connected with nutritional status, which has been reported to be altered in 14% to 25% of NET patients [64,65,66]. Particularly, patients with high-grade (G3) NET, progressive disease, and who are undergoing chemotherapy are at high risk of malnutrition (57.9%, 39.5%, and 42.3% respectively) [64].

Nutritional status in GEP–NET patients is deeply affected by the excessive production of gastrointestinal hormones, peptides, and amines, which could cause malabsorption, diarrhea, steatorrhea, and altered gastrointestinal motility. Furthermore, surgical resection of GEP–NET could change the anatomy of the gastrointestinal tract, and therapy with somatostatin analogs (SSAs) could modify the function of the gastrointestinal tract by inhibiting the secretion of pancreatic enzymes and hormones, impairing normal absorptive function (Figure 1) [19,67]. It has been recently demonstrated that GEP–NET patients have a dietary pattern with significantly lower adherence to the Mediterranean diet compared to a health-matched control group, less frequently consuming vegetables, fruits, wine, fish/seafood, and nuts, and more frequently red/processed meats, butter, cream, margarine, and soda drinks [20]. All these factors could affect the intake and absorption of vitamin D and calcium, which play key roles in the maintenance of bone health, with consequent bone loss. 

The role of vitamin D is reported in a separate section of this manuscript.

### 2.4. Therapies 

The surgical approach or medical treatment represents the main therapeutic options reported in current guidelines for GEP–NET, depending on tumor location, grade, size, and symptoms. Surgical removal of the primary tumor is the preferred treatment where it is possible, but it can also be considered in metastatic disease, and this may have survival benefits in some sites. 

Regarding pNET, enucleation, Whipple resection, or distal pancreatectomy/splenectomy are recommended in case of symptoms, intermediate-to-high grades (G2–G3), or measure greater than 2 cm. Conversely, if smaller than 2 cm, low-grade, and nonfunctioning, pNET can be monitored according to a “watch-and-wait” strategy [12,68]. Regarding small intestinal NET, partial small bowel resections are usually performed for jejunal or proximal ileal tumors, whereas right hemicolectomy is indicated for tumors arising in or near the ileocecal valve [69]. 

There are no data about a direct impact of surgical treatment on bone health in NET patients. Nevertheless, surgical treatment can influence nutritional status, causing diarrhea for several reasons. As previously discussed, the extent of the intestinal resection and the anatomical position of the resected region are the main factors with an impact on bowel transit time, vitamin B12 action, bile acid secretion, fat-soluble vitamins absorption and digestion [19,67,70].

Concerning medical therapy of GEP–NET, SSAs are the treatment of choice in patients with low grade G1–G2 tumor, in functional syndrome, if surgical resection cannot be performed or in case of metastatic disease [12]. The most common SSA side effects are steatorrhea, flatulence, nausea, and abdominal pain, all induced by inhibition of pancreatic digestive enzymes secretion and suppression of intestinal motility, regardless of dosage or formulation (octreotide or lanreotide) used [71]. SSAs may reduce intestinal fluid secretion and also the secretion of pancreatic enzymes and bile acids, which diminish fat absorption. Thus, similarly to surgical treatment, a direct relationship between SSA treatment and bone has not been demonstrated. It could be possible that malabsorption of fat and fat-soluble vitamins may lead to reduced bone density due to vitamin D deficiency [19]. Nevertheless, this hypothesis has been contradicted. The study from Motylewska et al. has not revealed any significant difference in 25-hydroxyvitamin D [25(OH)D] levels between patients with NET who were receiving or not SSAs, indicating that long-term SSA therapy does not affect serum vitamin D level. Furthermore, this study has shown that the average concentration of 25(OH)D did not depend on primary tumor localization. Thus, these results have not confirmed the hypothesis that patients with GEP–NETs may be a group of higher risk of vitamin D deficiency [29]. 

Patients with metastatic pNET have a larger number of other treatment options, with cytoreductive effects. First-line SSAs can be followed with everolimus and sunitinib, and peptide receptor-radionuclide therapy (PRRT) with radiolabeled synthetic and stable SSA (90Yttrium DOTATOC/DOTATATE or 177Lutetium DOTATOC/DOTATATE) [69,72].

Everolimus is an oral mTOR inhibitor. MTOR signaling has been shown to regulate osteoclastogenesis and osteoclast function [73]. A study in an osteotropic breast cancer model demonstrated that everolimus had a bone-protective efficacy both in vitro and in vivo, by impairing osteoclastogenesis and preventing the bone loss [74]. Moreover, everolimus has been associated with nausea, vomiting anorexia, stomatitis, and diarrhea—all factors indicating a malnourished status [71]. Sunitinib is an oral multitarget tyrosine kinases inhibitor (TKI), which mainly binds the vascular endothelial growth factor (VEGF) receptors and the platelet-derived growth factor (PDGF) receptors. In the literature, data about this drug class and bone metabolism are very scarce. However, it has been associated with an increased risk of osteonecrosis of the jaw in patients with bone metastatic renal cell carcinoma who were treated with bisphosphonates [75]. Sunitinib is also associated with diarrhea and possible other complications related to malnutrition and vitamin D malabsorption [71]. Cabozantinib is a new TKI that is currently under investigation in metastatic pNET. In a recent clinical trial on patients with advanced renal cell carcinoma and bone metastases, cabozantinib has been associated with an improvement of survival parameters when compared with everolimus. Furthermore, cabozantinib determined an important change in bone biomarkers, including decreases in the bone formation marker procollagen type 1 N propeptide (P1NP) and the bone resorption marker C-terminal telopeptide of type 1 collagen (CTx), but the authors did not provide an explanation for this [76]. 

PRRT is generally well-tolerated, but it is not completely free from side effects that can be acute or delayed. Acute side effects include nausea, vomiting, and headache, usually mild and self-limiting. Other acute adverse events are fatigue, abdominal pain, asthenia, and flushing [71]. To the best of our knowledge, at present, there are no studies about 177Lu DOTATATE or 90Y DOTATOC and bone metabolism. 

Recommended chemotherapy, as cytotoxic therapy, includes the following: the combination of cisplatin and etoposide, streptozocin (STZ) with 5-fluorouracil (5FU), temozolomide (TMZ) +/- capecitabine, oxaliplatin combinations with fluoropyrimidines (5-FU or capecitabine) and irinotecan-based therapy [77]. To our knowledge, all of these therapeutic agents may be associated with malnourished status, but their potential effect on bone has not yet been explored. 

In the clinical management of CS, a new drug has recently been introduced. Telotristat etiprate is an oral inhibitor of the enzyme tryptophan hydroxylase, which is the rate-limiting step in serotonin synthesis. The phase 3 TELESTAR study demonstrated that treatment with telotristat ethyl was generally well tolerated and was associated with significant reductions in bowel movement frequency and urinary 5-HIAA levels in patients with CS not adequately controlled by SSA therapy. However, this agent has no effect on tumor mass [78]. Common side effects of telotristat include nausea, abdominal pain, and a low rate of depression. We may speculate that, by reducing diarrhea, telotristat could ameliorate the nutritional status of NEN patients, but further studies are needed [79]. 

Therefore, GEP–NET treatment can have a role in determining disturbances of bone metabolism, but the kind of relationship between tumor, its treatment, and bone needs to be clarified. 

### 2.5. Impact of Quality of Life

Quality of life is an important measure of patients’ perception of the burden of the disease and the impact of the different treatment modalities. Several studies have reported a worse HRQoL in GEP–NET patients compared to matched controls [80,81]. Particularly, NET patients reported poorer physical function, sleep disorders, discomfort, and depression. Furthermore, patients with recurrent disease reported significantly higher anxiety, impaired overall physical function, impaired sleep, and significant fatigue compared to those with no current NET [82]. It has been reported that in patients with depressive disorder, the duration of the disease negatively correlates with BMD and physical activity, which, in turn, significantly reduces the HRQoL [83]. Moreover, low physical activity is associated with an increased risk of osteoporosis [84]. In addition, anxiety has been associated with BMD in the lumbar spine and femoral neck [85]. Therefore, depression, low physical activity, and anxiety worsen bone health. Although the impact of depression and low physical activity on bone health is not yet been investigated in GEP–NET, we speculate that these two factors may have an impact on bone metabolism in GEP–NET patients. However, further studies are needed to better establish this potential correlation.

## 3. Vitamin D in GEP–NET

Since its discovery, there has been an increasing understanding of the role of vitamin D [86]. It has long been known for regulating calcium and phosphate homeostasis and bone mineralization. Nowadays, vitamin D has been proven to have many pleiotropic actions. The expression of vitamin D-activating enzymes and vitamin D receptor (VDR) in pancreatic islets, heart, muscle, brain, gonads, prostate, breast, and immune cells suggests nonclassical effects related to local activation of vitamin D, including the regulation of immune system and influence on cellular growth and differentiation [87,88]. These nonclassical effects suggest a significant impact on pathogenesis and outcome of tumors, diabetes, cardiovascular, autoimmune, and infectious diseases [89,90,91,92,93,94,95,96]. 

Several studies have highlighted a correlation between vitamin D deficiency and tumor cell growth, with an impact on cell differentiation, apoptosis and adhesion, and angiogenetic and metastatic processes [97,98,99,100,101,102]. An in vitro study using RIN-m cells, a rat insulinoma cell line expressing VDR, demonstrated that the exposure to 1α,25-dihydroxyvitamin D3 [1α,25(OH)_2_D_3_] or MART-10, a 1α,25(OH)_2_D_3_ analog, inhibits cell growth by induction of cell-cycle arrest at G0/G1 phase and apoptosis [103]. The same group also demonstrated that both MART-10 and 1α,25(OH)_2_D_3_ effectively inhibited VEGF-induced metastatic potential of RIN-m cells through reducing the epithelial–mesenchymal transition and F-actin, which plays a role in cell migration and invasion [104]. These studies suggest that vitamin D and its analogs might represent a promising regimen for the treatment of NET patients. 

As mentioned above, GEP–NET patients have an increased risk of malnutrition due to tumor hormonal secretion and treatments, which affect the absorption of nutrients, including vitamin D [19]. Vitamin D deficiency, defined as 25(OH)D levels ≤20 ng/mL (≤50 nmol/L) and insufficiency, defined as 25(OH)D levels ranging from 20 to 29.9 ng/mL (52–72 nmol/L) [105], is described in between 46% and 81% of patients with NET (Table 1) [21,22,29,30]. This large range observed in the prevalence of vitamin D deficiency or insufficiency could be due to several factors, including latitude of the country, seasons and different assays used for the measurements of vitamin D levels [106,107]. Lind et al. found a high prevalence of low BMD (82% of cases) and of vitamin D and B12 deficiency (74% and 32% of cases, respectively) in a cohort of 50 patients with small intestinal NET [21]. In a recent study, Massironi et al. found vitamin D deficiency in 33% and insufficiency in 68% of cases in an Italian population of 138 GEP–NET patients [22]. No differences in vitamin D levels were observed between pNET and other tumor sites, as well as no difference when considering tumor grades and staging [22]. Similarly, a more recent study by Robbins et al. evaluating 183 patients with GEP–NET showed a prevalence of vitamin D deficiency/insufficiency in 63% of cases [30]. 

The influence of medical treatment with SSAs on vitamin D levels is controversial. In the Italian study, it was highlighted that there were lower vitamin D levels in GEP–NET patients treated with SSAs compared to those not treated by SSAs [22]. On the contrary, the other two studies did not find any differences in vitamin D levels between NET patients with or without SSA treatment [29,30]. 

It has been observed that vitamin D could influence the clinical outcome of patients with different malignant tumor types, reducing overall mortality and disease progression [108,109]. Interestingly, also in GEP-NET patients, an inverse correlation has been observed between vitamin D and clinical outcome, considering both overall survival and progression-free survival [22]. Particularly, the correlation between vitamin D and overall survival was also significant in multivariate analysis. However, these data were not confirmed in the later study by Robbins et al., who did not find a correlation between vitamin D and parameters of tumor progression [30]. Despite these contrasting results, both studies demonstrated that vitamin D supplementation with over-the-counter vitamin D preparations improves vitamin D levels in most GEP–NET patients. In the study by Robbins et al., it has been reported that, during the observation period of 24 months, the supplemented vitamin D leads to a significant improvement of vitamin D status [30]. However, the observation period of this study was too short to evaluate disease progression or mortality, considering that GEP–NET progress slowly. In the study referenced above, Massironi et al. collected 5 years of data and showed an improvement in overall survival in patients with GEP–NET who had received replacement vitamin D [22]. 

In conclusion, few studies have evaluated vitamin D status in GEP–NET patients and they have focused on the differences in clinical outcomes between patients with insufficient/deficient levels of vitamin D vs. patients with sufficient vitamin D levels (Table 1). Only one study also reported the incidence of low BMD; however, without correlating BMD value with vitamin D levels [21]. Further studies are needed to better elucidate the role of vitamin D in GEP–NET and to investigate a relationship between vitamin D, BMD, and osteoporosis in GEP–NET patients. 

## 4. GEP–NET Associated to MEN1 and Bone Health 

Most GEP–NET are sporadic, but in approximately 10% of cases, they occur as part of familial syndromes, such as MEN1. MEN1 is a rare genetic syndrome characterized by the onset of different endocrine as well as non-endocrine neoplasms. Patients with MEN1 usually develop parathyroid adenomas and primary HPT (90%–100% of cases), GEP–NET which are usually multiple and located in the pancreas and duodenum, and can either be functional (gastrinoma is the most common) or nonfunctional (80%–100%), and anterior pituitary adenomas (20%–60%). Less commonly, patients present with tumors in the lungs, thymus and adrenal glands, as well as lipomas, angiofibromas, and collagenomas. MEN1 is inherited in an autosomal dominant pattern with high penetrance and is caused by more than 1500 different mutations of the *MEN1* tumor suppressor gene located on chromosome 11q13, which encodes the protein menin [110,111,112]. Menin is involved in various biological functions in several tissues, even in bone, although the precise mechanisms by which menin acts as a tumor suppressor still remains unclear. Menin can interact directly with VDR and enhances the transcriptional activity of VDR. Menin also shows interactions with other bone-related factors, such as retinoblastoma protein, estrogen receptor, Hox and heat shock proteins, insulin-like growth factor-binding protein-2, and telomerase. Furthermore, menin is important for both early differentiation of osteoblasts and inhibition of later differentiation [113,114]. In recent years, our knowledge of the genetic mutations in GEP–NET has expanded considerably, but unfortunately, no close genotype-phenotype correlation has been identified in MEN1. This syndrome can affect all age groups, and more recently, the rate of complications associated with MEN1-related morbidity and mortality have declined, due to early diagnosis and improvement of therapeutic approaches. Death in patients with MEN1 was previously attributed to the consequences of excess gastric acid secretion, but, nowadays, metastatic GEP–NET represents the leading cause of death [115]. 

Alterations of bone metabolism in patients with MEN1 and GEP–NET are mainly due to the frequent coexistence of HPT in the syndrome. According to current MEN1 guidelines, patients with gastrinoma or multiple pancreatic NET at any age should be tested for MEN1 and therefore screened for HPT. HPT due to parathyroid hyperplasia is the most frequent and usually the earliest clinical manifestation of MEN1. Although it is not closely related to mortality, it is responsible for morbidity, as bone and renal complications are common. Patients may be asymptomatic and diagnosed with HPT due to incidental finding of elevated serum calcium or parathormone levels, but nephrolithiasis, renal colic, or even renal failure may be present at onset [110,115,116]. The same signs and symptoms related to sporadic chronic hypercalcemia are also present in MEN1 patients, and there is increased bone resorption and fracture risk. Unlike its sporadic counterpart, MEN1 related HPT occurs at an earlier age (33 years of age vs. 63 years) and characteristically shows multiple gland involvement. Surgery is the treatment of choice for MEN1 HPT, although the most appropriate timing and surgical approach are debated, and undoubtedly there is a higher persistence and recurrence rate compared with sporadic disease [117,118]. Nephrolithiasis and early bone mineral loss are frequent, extensive, and progressively worsen during the course of the disease. Interestingly, patients with long-standing HPT (>10 years) and gastrinoma associated with HPT have shown significantly lower BMD values in the distal radius [31]. Regarding the association of GEP–NET and HPT, patients presenting with both gastrinoma and HPT have been reported to have a tendency for higher PTH levels and a higher percentage of urolithiasis than in patients without gastrinoma. It has been supposed that the early age of onset of HPT in MEN1 interferes with the achievement of peak bone mass. Furthermore, the finding of low levels of 25(OH)D may worsen the BMD status and increase PTH levels (Table 1) [31]. The association of gastrinoma with Zollinger Ellison Syndrome (ZES) and a more aggressive form of HPT with a high rate of nephrolithiasis, persistence, and recurrence of HPT has been described, and these data support the need of strict follow-up in these patients [119]. Importantly, the loss of BMD is greater in MEN1 related HPT, compared to sporadic primary HPT and occurs both in the cortical and trabecular bones [120]. It has been reported that one year after parathyroid surgery, there is a recovery of decreased bone mineral density in the lumbar spine and femur, suggesting these two sites to evaluate outcomes (Table 1) [32]. In another study, one year postoperatively, MEN1 HPT patients had improved Z-scores at the lumbar spine, whereas the sporadic group had improved Z-scores at the lumbar spine, total hip, and femoral neck, suggesting a better recovery of BMD of sporadic patients compared with MEN1 (Table 1) [33].

Cinacalcet, a calcimimetic that modulates the calcium-sensing receptor, reduces PTH secretion and lowers serum calcium and phosphate concentration and is safe and effective in MEN1 HPT as a secondary choice of treatment. Nevertheless, no significant changes have been observed in either trabecular or cortical BMD, and no changes in bone turnover markers have been detected after one year of treatment [34].

Patients with GEP–NET, especially if located in the pancreas and duodenum, presenting with disturbance of bone metabolism, should be carefully evaluated, as MEN1 and other genetic syndromes account for 10% of GEP–NET. Disturbance of bone metabolism and vitamin D alteration in patients with GEP–NET and MEN1 are mainly related to the coexistence of HPT, but the association of gastrinoma and ZES seem to worsen HPT. Furthermore, the long-term treatment of gastrinoma with proton pump inhibitors may moderately increase the risk of any-site, hip, or spine fracture [121,122]. Long-term follow-up studies, including large populations, are needed to increase the knowledge of bone metabolism and recoverability in MEN1.

## 5. Clinical Management of Bone Health and Vitamin D Supplementation in GEP–NET

GEP–NET patients seem susceptible to developing osteopenia and osteoporosis. As mentioned above, only one study demonstrated low bone density in NET patients [18], while several studies described a considerable percentage of vitamin D deficiency and insufficiency [21,22,29,30] in this category of patients. 

**Figure 4:**
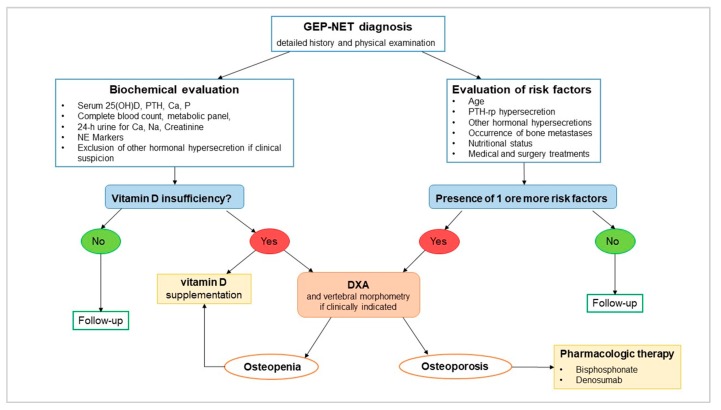
Algorithm for the management of bone metabolism disturbances in GEP–NET. Abbreviation: Ca, calcium; DXA, dual X-ray absorptiometry; Na, sodium; NE, neuroendocrine; P, phosphate; PTH, parathyroid hormone; 25(OH)D, 25-hydroxyvitamin D.

Thus, data from literature are too scarce to highlight the necessity of specific guidelines for the diagnosis and clinical management of disturbances of bone metabolism in GEP–NET, and, in clinical practice, the lack of specific guidelines is evident. GEP–NET patients have several factors, such as hormonal hypersecretion, miRNAs expression, nutritional status, medical and surgical treatments, vitamin D deficiency, worsening quality of life, and aspects correlated to MEN1 that can affect bone health. In addition, laboratory studies showed that vitamin D might represent a key element in malignant neoplasm pathophysiology, with a potential therapeutic role. For all these reasons and also because osteoporosis can have important social and economic implications, an algorithm with some diagnostic and therapeutic indications in GEP–NET may be useful (see Figure 4).

All GEP–NET patients should undergo clinical assessment for osteoporosis and fracture risk, including a detailed history and physical examination. Risk factors that should be considered are age, hormonal hypersecretion, nutritional status, and history of medical and surgical treatments (Figure 4). Laboratory evaluation should include measurement of serum 25(OH)D in all patients. Then, a complete blood count, a comprehensive metabolic panel, intact PTH, phosphate, and a 24-hour urine collection for calcium, sodium, and creatinine should be performed. Also, the measurement of neuroendocrine markers that could be associated with a specific tumor type is suggested. If clinical suspicion is present, hormonal hypersecretion should be excluded. 

In the case of vitamin D insufficiency and/or in the presence of risk factors, then dual-energy X-ray absorptiometry (DXA) is recommended for assessing BMD [123]. Central DXA measurement is recommended at the lumbar spine (L1-L4) and proximal femur; in the case of hyperparathyroidism, forearm measurement is recommended. Diagnosis of osteoporosis is be based on presence of fragility fractures in the absence of other metabolic bone disorders or on the presence of a T-score of –2.5 standard deviation (SD) or lower in the lumbar spine (anteroposterior), femoral neck, total hip, and/or one-third radius even in the absence of a prevalent fracture. A T-score between –1.0 and –2.5 SD indicates osteopenia [123]. In the case of risk factors, the presence of vertebral fractures should be evaluated by vertebral morphometry [124]. 

Pharmacologic therapy is recommended in patients with osteoporosis or with osteopenia and multiple risks of fracture. Current recommendations suggest vitamin D supplementation to reduce mortality associated with malignant neoplasms [125]. This positive effect was also demonstrated in GEP–NET [22]. A very recent meta-analysis showed that circulating levels of 25(OH)D of 54–135 nmol/L (21.6–54 ng/mL) may contribute to reducing cancer mortality [125]. However, evidence coming from randomized clinical trials suggest 25(OH)D concentration above 75 nmol/L (30 ng/mL) as optimal levels [93,125]. To achieve this concentration, the Endocrine Society guidelines suggest an intake of 1000 to 2000 international units (IU)/day of vitamin D3 (cholecalciferol) for adults with vitamin D insufficiency [105]. Higher doses may be necessary in the presence of certain factors such as diarrhea and malabsorption [126]. Moreover, optimal vitamin D levels are necessary also to prevent bone damage [126]. 

Different approved agents, including alendronate, risedronate, zoledronic acid, and denosumab, have been shown to be efficacious in reducing hip, non-vertebral, and spine fractures and are appropriate as initial therapy for most patients at risk of fracture [127]. Patients with lower or moderate fracture risk can be started on oral agents. Patients with gastrointestinal problems that might not absorb oral medications should take injectable agents such as denosumab or zoledronic acid [128]. Denosumab is a human monoclonal antibody that prevents receptor activator of nuclear factor kappa-B ligand (RANKL) from binding to its receptor, RANK, thereby reducing the differentiation of precursor cells into mature osteoclasts and decreasing the function and survival of activated osteoclasts. In postmenopausal women, the anti-fracture efficacy of denosumab has been documented for vertebral fractures, hip fractures and non-vertebral fractures (−68%, −40% and −20%, respectively, over three years of therapy). Moreover, evidence has shown that denosumab also has efficacy in the treatment of patients with breast cancer treated with aromatase inhibitors and men on androgen deprivation therapy for prostate cancer [129]. 

Follow-up of patients should ideally be conducted in the same facility with the same machine. DXA should be repeated every 1 to 2 years until findings are stable [129]. Due to the complexity in the management of osteopenia/osteoporosis in patients with malignant neoplasms, bone assessment and clinical management in GEP–NET patients need a multidisciplinary approach. 

## 6. Discussion and Conclusions

Bone is a highly dynamic organ that undergoes significant constant remodeling balanced by osteoblastic bone formation and osteoclastic bone resorption. Disturbance of this equilibrium leads to skeletal diseases, including osteoporosis [2]. Osteopenia and osteoporosis have been described in a large percentage of GEP–NET patients (up to 76% of cases) [18,21,25,26], and an increased risk of lower BMD is particularly pronounced in younger patients (<50 years old) [18]. This association with low BMD observed in GEP–NET patients could be explained by the fact that, besides bone metastasis [13], GEP–NETs are associated with several factors that could interfere with the normal bone homeostasis, including hypersecretion of substances biologically active on bone tissue, miRNA expression, nutritional status, which in turn could be affected by medical and surgical treatments, vitamin D deficiency, worsening quality of life and aspects correlated to MEN1 [21]. Among the hormones that could be secreted by functioning GEP–NET, 5-HT, cortisol and PTH-rp could play a major role in affecting bone metabolism [28,41,48,53]. Nutritional status in GEP–NET patients is deeply affected by the excessive production of gastrointestinal hormones, surgical resection changing the anatomy of the gastrointestinal tract, and treatment with SSA and chemotherapy. All these factors cause malabsorption with a consequent deficit of vitamin D and other electrolytes involved in bone metabolism [19,22,70]. Vitamin D deficiency is highly prevalent in GEP–NET patients [21,22,29,30]. Since vitamin D plays a central role in bone metabolism [86], its deficiency may negatively impact bone health in GEP–NET patients. However, studies focusing on vitamin D and bone in GEP–NETs are missing. Weak or indirect effects on bone are described for depression, low physical activity, as well as specific miRNAs (miRNA-210, -21 and -196a) [56,57,83,130]. Finally, specific aspects of MEN1 syndrome, including primary HPT and gastrinoma, have been shown to worsen BMD. Moreover, the protein menin has a direct effect on VDR and other bone-related factors, as well as playing a central role in the differentiation of osteoblasts [113,114].

Although patients with GEP–NETs usually have good long-term survival, they have a greater clinical burden of disease than healthy controls and are at high risk of presenting with osteoporosis at a younger age. Osteoporosis, together with other comorbidities, may negatively impact the quality of life of these patients and could contribute to higher medical expenses. Due to the lack of scientific evidence, there are no specific guidelines designed to regulate the diagnosis and clinical management of disturbances of bone metabolism in GEP–NETs. Bone assessment and the pathological exams in NET needs a multidisciplinary approach. We suggest supplementation with vitamin D in those patients who have insufficient or deficient vitamin D levels since it has been demonstrated that supplementation does improve the vitamin D levels and may correlate with better clinical outcomes. Moreover, optimal vitamin D levels are also necessary to prevent bone damage. Finally, we recommend screening with a bone density scan in those patients who present with more than one factor that could be associated with bone loss. Further studies are urgently needed to better delineate the relationships and associations between these interesting areas of medicine and science.

## Figures and Tables

**Table 1:** Principal clinical studies evaluating bone metabolism and vitamin D in GEP–NET.

Author, Year and Reference	Type of Study	Number of Patients	Principal Aim	Principal Findings Associated with Bone Metabolism or Vitamin D
Hess et al. 2012 [18]	Retrospective case-control.	1762 NET and 3524 controls (1:2 ratio).	Comorbidities in NET.	Adjusted risk of osteoporosis/osteopenia was higher in NET patients among those 50 years or younger.
Van Dijk et al. 2012 [24]	Retrospective.	84 carcinoid patients: 61 with increased 5-HT levels and 23 with low 5-HT levels (controls).	Differences in markers of bone metabolism between the two groups.	No significant differences in markers of bone metabolism between hyper-secretors and controls.
Sen Gupta et al. 2014 [25]	Observational.	46 consecutive NET.	Relationships between urinary 5-HIAA and BMD measured by DXA	41.3% had osteoporosis and 32.6% osteopenia. Urinary 5-HIAA was not an independent predictor for BMD.
Walsh et al. 2013 [26]	Cross-sectional.	25 patients with carcinoid syndrome and 25 healthy controls.	Association of carcinoid syndrome with bone formation markers, BMD and bone structure.	No measures of bone density or bone structure differed significantly between cases and controls.
Byun et al. 2017 [27]	Case report.	Case of pNET producing ACTH.	Descriptive.	40-year-old female patient with ophthalmologic discomfort, osteoporosis, and hypokalemia with diagnosis of pNET.
Dobnig et al. 1996 [28]	Case report.	Case of appendix carcinoid producing ACTH-	Descriptive.	34-year-old female patient with multiple spontaneous rip fractures and T-score at lumbar spine -4.3 SD. Increasing of BMD after tumor resection.
Lind et al. 2016 [21]	Intervention.	50 consecutive SI-NET (25 controls and 25 supplemented with vitamin D, B12 and calcium).	DXA and gastrointestinal disorders.	Control group: 46% vitamin D deficiency and 76% low BMD. Supplemented group: 28% vitamin D deficiency and 60% low BMD. Vitamin D supplementation avoid severe deficiency.
Massironi et al. 2017 [22]	Observational.	138 GEP–NET.	Impact of vitamin D and OS and PFS.	68% cases had vitamin D deficiency. Patients treated with SSA had lower vitamin D levels. At multivariate analysis, vitamin D levels significantly correlate with OS.
Motylewska et al. 2016 [29]	Observational.	36 NET and 16 healthy controls.	Evaluation of vitamin D levels between the two groups.	No significant difference in vitamin D levels between NET and controls. SSA therapy did not aggravate vitamin D deficiency.
Robbins et al. 2018 [30]	Longitudinal, intervention.	183 GEP-NET.	Effect of vitamin D treatment after 2 year of follow-up.	Vitamin D insufficiency decreased from 66.6% at baseline to 44.9% and 46.2% after 12 and 24 months, respectively. Previous abdominal surgery predicted vitamin D levels.
Lourenco et al. 2010 [31]	Cross-sectional.	36 MEN1 patients with HPT.	Outcome of bone and renal complications.	Patients with long-standing HPT (>10 years) and gastrinoma/HPT presented significantly lower 1/3DR BMD values.
Coutinho et al. 2010 [32]	Case series.	16 HPT/MEN1.	Impact of total PTx on BMD in patients with HPT/MEN1.	BMD improvement in the lumbar spine, femoral neck, and total femur after 15 months from PTx.
Silva et al. 2017 [33]	Retrospective.	14 HPT/MEN1 and 104 sporadic HPT.	Impact of total PTx on BMD in patients with HPT/MEN1 *vs* sporadic HPT.	At baseline, HPT/MEN1 had significantly lower Z-score at lumbar spine, total hip, and femoral neck than sporadic HPT. 1 year after PTx, HPT/MEN1 showed a better Z-score only at lumbar spine compare to baseline.
Giusti et al. 2016 [34]	Longitudinal, intervention.	33 MEN1.	Effect of cinacalcet on HPT.	No significant changes in BMD, and bone turnover markers after 1 year of treatment

Abbreviation: BMD, bone mineral density; HPT, primary hyperparathyroidism, DXA, dual X-ray absorptiometry; MEN1, multiple endocrine neoplasia type 1; NET, neuroendocrine tumor; GEP–NET, gastro-entero-pancreatic NET; OS, overall survival; PFS, progression-free survival; pNET, pancreatic NET; PTx, parathyroidectomy; SI-NET, small intestinal NET; SSA, somatostatin analog; 1/3DR, proximal one-third of the distal radius; 5-HIAA, urinary 5-hydroxy-indoleacetic acid; 5-HT, serotonin.
